# Machine learning on a smartphone-based CPT for ADHD prediction

**DOI:** 10.3389/fpsyt.2025.1564351

**Published:** 2025-11-07

**Authors:** Núria Casals, Simon Larsson, Mikkel Hansen

**Affiliations:** Medical Department, Qbtech AB, Stockholm, Sweden

**Keywords:** ADHD, machine learning, CPT, smartphone, mobile, motion sensor, face tracking, AI

## Abstract

**Objectives:**

Continuous Performance Tests (CPTs) are widely utilized as objective measures in the assessment of Attention-Deficit/Hyperactivity Disorder (ADHD). The integration of sensor data in smartphones has become increasingly common as a way of monitoring several behavioural indicators of mental health. Machine learning has started being utilized in the field of ADHD to improve diagnosis. This investigation explores (i) the feasibility of using smartphone devices to administer a CPT for ADHD assessment and (ii) whether data from built-in sensors in smartphone devices is useful for predicting a diagnosis.

**Methodology:**

The study uses data from a control group of neurotypical individuals and an ADHD cohort of unmedicated patients. The dataset is divided into a training and test set, and a machine learning model is developed using the training set. The model is trained by dividing features into four groups, Demographic, CPT, Face, and Motion, which are then sequentially added and evaluated on their ability to predict ADHD.

**Results:**

A total of 952 neurotypical individuals and 292 unmedicated ADHD patients were part of the study. The best performing model combines all feature groups by a sensitivity of 0.808, specificity of 0.795 and area under the precision-recall curve (PR-AUC) of 0.799, with a considerable performance increase due to the phone sensor features addition. Results did not differ significantly by age group (6–11 and 12–60 years old) and sex.

**Conclusion:**

The study provides a robust machine-learning model that is based on a large control group together with an ADHD cohort. The experiments demonstrated that ADHD can be assessed with high accuracy using CPTs on smartphones. Integrating face-tracking and motion sensor data with CPT features further enhanced performance, indicating that data from a smartphone device can surpass the accuracy of traditional computer-based ADHD assessments.

## 1 Introduction

Attention-Deficit/Hyperactivity Disorder (ADHD) is a neuro-developmental disorder with symptoms of inattention, hyperactivity and impulsivity greater than expected for their age or developmental level (1). Assessment of ADHD is a complex diagnosis process for several reasons (2), including:

- Time consuming. Early diagnosis makes it possible to contemplate and implement suitable treatment strategies. A survey on French children found that on average, the time between the start of symptoms and ADHD diagnosis is longer than 4 years (3).
- Subjective measures. ADHD diagnosis is influenced by the perceptions of many different members of a child’s community. A lack of clear understanding of ADHD and the importance of its diagnosis and treatment still exists among many members of the community including parents, teachers, and healthcare providers (4). Objective data should also contribute to the clinical diagnosis of ADHD (5).

Overall, reliable testing that utilizes objective measures to assess the diagnosis of ADHD is needed. The current investigation is part of the development of a smartphone application (QbMobile) and aims to evaluate the performance of a machine learning model, by assessing (i) the feasibility of using smartphone devices to administer Continuous Performance Tests (CPTs) for Attention-Deficit/Hyperactivity Disorder (ADHD) assessment and (ii) whether data from the built-in motion sensors can be useful in making a diagnosis. The study will explore the impact of using a large control group together with new features that can be extracted from a smartphone device by using a machine learning model to recognize symptom patterns and predict the diagnosis.

## 2 Background

### 2.1 CPT and ADHD

CPTs are widely utilized as objective measures in the diagnosis of ADHD due to their ability to systematically evaluate attention and impulsivity. Unlike subjective assessments, such as behavioral rating scales and clinical interviews, CPTs provide quantifiable data on an individual’s cognitive functioning. These tests are designed to measure the individual’s attention and impulsivity during a sustained period, two critical areas often impaired in individuals with ADHD (6).

CPTs vary in their implementation, but a CPT involves presenting a series of stimuli. The participant must perform an action when a target stimulus appears and withhold the action for non-target stimuli. Performance is evaluated by looking at key measures such as:

- Omission Errors: Failing to respond to a target stimulus, indicating inattention.
- Commission Errors: Responding to a non-target stimulus, reflecting impulsivity.
- Correct Responses: The number of accurate responses to target stimuli.
- Response Time: Time taken to respond to target stimuli.
- Response Time Variability: Fluctuation in response times.

A systematic review of the utility of CPT among adults with ADHD showed an elevated risk of bias and substantial heterogeneity among the studies and while numerous studies reported differing scores between adults with ADHD and comparison groups, the findings were inconsistent (7). However, when excluding studies with small sample size, the CPT performance improves (8). Overall, it is agreed that CPT tests cannot be a substitute for subjective behavioral interviews, observations, and other clinical assessments, but they may serve as a valuable supplementary tool in the diagnosis of ADHD for both children and adults (9).

### 2.2 Face tracking and motion sensor-based data in psychiatric disorders

The integration of sensor data in smartphones has become more prevalent and the use of smartphones is an unobtrusive way of monitoring several behavioral indicators of mental health (10). Sensor-based data refers to quantitative information captured by phones through their embedded sensors. Modern smartphones are equipped with a variety of sensors, cameras with face tracking, accelerometers, gyroscopes, magnetometers, GPS, and biometric sensors like temperature.

Other objective measures are being used to complement CPTs in ADHD assessment. QbTest combines a CPT with measures of hyperactivity by performing face tracking using sensor data from an infrared camera and a motion capture marker attached to the head of the participant which has been shown to be effective in ADHD assessment (11).

There are currently no studies associating smartphone motion sensor data with ADHD, but recent studies reported that data collected from smartphone motion sensors can be associated with symptoms of schizophrenia, bipolar disorders, and depression. However, despite these associations, their usability in clinical settings for supporting therapeutic interventions has not yet been fully assessed and requires more thorough scrutiny (12).

A correlation has been found between depression scales and sensor data coming from GPS, accelerometer, gyroscope, microphone, and light sensor (13). It has also been concluded that sensor data can be associated with changes in depression, stress, and subjective loneliness over time (10). Another study used GPS, accelerometer, gyroscope, microphone, and phone calls to detect early changes in the state of a bipolar disorder patient (14).

### 2.3 Machine learning in ADHD

Machine learning algorithms use a range of statistical, probabilistic, and optimization methods to learn and identify valuable patterns within large, unstructured, and complex datasets (15).

Machine learning is increasingly being used in ADHD to improve diagnosis (16). By analyzing large datasets, machine learning algorithms can identify patterns and markers that may be indicative of ADHD symptoms, improving diagnostic accuracy and early detection (17).

One application is to use a machine learning model to learn correlations between ADHD diagnosis and answers from the ADHD symptoms rating scales such as Conners’ Adult ADHD Rating Scales (18, 19) and EarlyDetect (20).

Such models can also be applied to CPT tests like QbTest (21), Test Battery for Attention Performance (TAP) (22) and MOXO-CPT (23). Machine learning has also been used to link other kinds of objective measures to ADHD symptoms such as pupil diameter (24), event related potentials (ERPs) (25), serotonin transporters and genotypes (26), eye tracking (27) and magnetic resonance imaging (MRI) (28).

## 3 Methodology

### 3.1 Participants and procedure

A subset of data originated from two observational studies, a normative study and a study with patients being assessed for ADHD (performed in United States, Germany, the Netherlands, and the United Kingdom), was used for analyses in the present machine learning experiment. Participants between 6–60 years were included.

An ADHD cohort of 292 unmedicated participants were included and recruited through the research facility’s ADHD database. A pre-screening process via an online questionnaire was utilized and eligibility was confirmed by the research members at the participating sites, prior to participants’ engagement in the study. The neurotypical group consisted of 1244 individuals. The neurotypical group was selected based on the absence of any documented or suspected current or lifetime diagnosis of ADHD. It excluded anyone who had a concurrent medical diagnosis that could significantly affect test performance (i.e., brain injuries, Parkinson’s disease, current epilepsy or active seizures, amyotrophic lateral sclerosis (ALS), multiple sclerosis, dementias (e.g., vascular dementia, Alzheimer’s disease), psychiatric illness, etc.

To evaluate model performance, the dataset was divided into training and test sets, using an 80/20 split (29). Stratification was applied based on ADHD diagnosis, age group (children: 6–11 years; adults: 12–60 years), and sex to ensure balanced representation across these categories in both subsets.

Model selection was performed using a 5-fold cross-validation. That means that the training dataset is divided into five equal parts, or “folds”. The model is trained on four folds and tested on the remaining one. This process is repeated five times, each time with a different fold serving as the validation set. The same stratification criteria—ADHD diagnosis, age group, and sex—used in the training/test split were consistently applied during the 5-fold cross-validation process. The stratification ensures that each fold maintains a balanced representation of these categories, reducing the risk of randomness introducing skewed distributions and providing a more robust and reliable evaluation of the model’s performance. The results are then averaged to provide an overall performance metric. Training and testing the model on different subsets of the data helps to minimize overfitting and provides a more accurate estimate of how the model will perform on the test set (30).

### 3.2 Measures

#### 3.2.1 Demographic features

To account for variables outside the test setting that could influence ADHD diagnosis, several demographic features were incorporated as control measures. Sex was added to make up for the fact that sex differences, although minor, have been observed in ADHD prevalence (31). Similarly, age was added because the expression of ADHD symptoms has been shown to vary with age (32). Furthermore, the relative age effect, where younger children in a class are more frequently diagnosed with ADHD compared to their older peers (33, 34), because of this birth month was added as a demographic feature. These measures aim to quantify the effect of demographic factors in the data and subsequent model.

#### 3.2.2 CPT features

A CPT test on a smartphone device was used for the study where participants responded by tapping the screen. The stimuli were shown 200 milliseconds in a two-second interval for 10 minutes. The test objective was different depending on the age group, but the test duration was kept constant to ensure comparability in sustained attention measures while minimizing participant burden.

For the adult test, the presented stimuli are a blue circle, a blue square, a red circle, and a red square. The phone screen needs to be pressed when two identical stimuli are shown in a row. The children’s test stimuli are a gray circle and a gray circle with a cross in random order of appearance. The phone screen must be pressed when the gray circle appears.

#### 3.2.3 Face tracking features

Apple’s ARKit (35) was employed for real-time tracking of the participant’s face position in 3 dimensions during the execution of the CPT. The resulting time series data was subsequently processed to extract features that captured the participant’s activity level and movement patterns throughout the test duration.

#### 3.2.4 Motion sensor features

The smartphone’s integrated motion sensors were utilized to monitor the participant’s movements while they held the device during the CPT. The accelerometer captured linear acceleration across three axes (x, y, and z), and the gyroscope measured rotational motion in terms of pitch, roll, and yaw. The time series data collected from each test was processed to generate a set of features aimed at capturing the activity and movement patterns observed during the test.

### 3.3 Model

The predictive model used was LightGBM (36) which is a form of gradient boosting machine (37) where a sequence of decision trees (38) where each subsequent tree attempts to correct the error of the previous one.

### 3.4 Evaluation

The final model is evaluated on the test set. The primary evaluation metric, also used as the optimization criterion for model selection, is the area under the precision-recall curve (PR-AUC). PR-AUC is widely applied in evaluating diagnostic test accuracy (39), as it is especially informative for class-imbalanced predictive tasks due to its sensitivity to changes in false positive rates (40).

Alongside PR-AUC, sensitivity and specificity were evaluated as they are standard metrics for reporting accuracy in medical classification tasks (41). Sensitivity measures the model’s ability to correctly identify positive cases, while specificity assesses its ability to correctly identify negative cases.

## 4 Results

**Tables 1**, **2** show the sizes of the neurotypical, ADHD cohorts and their respective distribution in the train and test sets. The used dataset had 1244 tests, and the 80/20% train-test split resulted in a train set of 997 and a test set of 247 tests. In total, the sample had 292 ADHD and 952 neurotypical individuals. Regarding the age and sex distribution, there were 1104 adults and 140 children, 718 of them were female and 526 male.

**Table 1:** Participant cohorts.

Split	ADHD (n)	Neurotypical (n)	Total (N)
Train	229	768	997
Test	63	184	247
Total	292	952	1244

**Table 2:** Participants by age and sex groups.

Split	Adult (n)	Child (n)	Female (n)	Male (n)
Train	891	106	572	425
Test	213	34	146	101
Total	1104	140	718	526

**Table 3** contains the contribution of the feature groups to ADHD prediction. It shows the results of the model evaluated on the test dataset. To ensure robustness and reliability, the performance is reported as the average and standard deviation across 10 independent trainings of each model. The machine learning model shows no inherent bias in the data associated with the Demographic features, as evidenced by its poor performance when using only these features. The model achieves a low PR-AUC of 0.327, indicating a lack of class separation.

**Table 3:** Incremental contribution of feature groups. Values are reported as mean (standard deviation) over 10 independently trained models.

Features	Sensitivity	Specificity	PR-AUC
Demographic	0.935 (0.054)	0.190 (0.156)	0.327 (0.019)
Demographic + CPT	0.646 (0.243)	0.620 (0.199)	0.614 (0.034)
Demographic + CPT + Face	0.775 (0.091)	0.652 (0.137)	0.689 (0.026)
Demographic + CPT + Face + Motion	0.808 (0.050)	0.795 (0.052)	0.799 (0.023)

**Table 4** reports the one sided t-test results where the null hypothesis is that adding a new feature group does not significantly increase the PR-AUC. In all three cases the null hypothesis was rejected with a p-value< 0.001. In consequence, the addition of CPT, Face and Motion feature groups did significantly increase the PR-AUC of the resulting model.

**Table 4:** Results of one-sided t-tests evaluating the significance of PR-AUC improvements with the addition of new feature groups, presented with corresponding t-statistics and p-values.

Features	Adding CPT	Adding face	Adding motion
t-statistic	23.246	5.450	10.205
p-value	<0.001	<0.001	<0.001

The best-performing model combined all feature groups achieved a PR-AUC of 0.799, sensitivity of 0.808 and specificity of 0.795. **Tables 5**, **6** show the performance of the best-performing model split by age and sex groups reporting mean, standard deviation and 95% confidence interval computed via bootstrapping. These results indicate good overall performance and robustness across confidence intervals and demographic subgroups, though a slight class imbalance is reflected in lower specificity for children.

**Table 5:** Age and sex split of performance results.

Group	Features	Sensitivity	Specificity	PR-AUC
Adult	Demographic + CPT + Face + Motion	0.752 (0.059)	0.810 (0.050)	0.731 (0.035)
Child	Demographic + CPT + Face + Motion	0.987 (0.040)	0.658 (0.116)	0.957 (0.020)
Female	Demographic + CPT + Face + Motion	0.867 (0.057)	0.773 (0.044)	0.800 (0.027)
Male	Demographic + CPT + Face + Motion	0.730 (0.064)	0.827 (0.073)	0.800 (0.031)

Values are reported as mean (standard deviation) over 10 independently trained models.

**Table 6:** 95% confidence interval of the model demographic + CPT + face + motion for sensitivity, specificity, and PR-AUC.

Group	Sensitivity	Specificity	PR-AUC
Adult	[0.717, 0.788]	[0.777, 0.840]	[0.710, 0.752]
Child	[0.960, 1.000]	[0.589, 0.737]	[0.944, 0.968]
Female	[0.828, 0.903]	[0.744, 0.799]	[0.785, 0.818]
Male	[0.693, 0.770]	[0.782, 0.869]	[0.780, 0.818]
Total	[0.778, 0.837]	[0.761, 0.826]	[0.785, 0.814]

## 5 Discussion

Our results supported the study’s hypothesis, validating the capability of a machine learning algorithm to predict ADHD diagnoses using a smartphone device. It confirmed (i) the feasibility of performing CPT tests in a smartphone device and (ii) the positive impact of sensor data on the performance of the tests. These findings align with prior research emphasizing the utility of smartphone technology in mental health diagnostics while offering a novel contribution by integrating sensor data to improve predictive accuracy (42).

The model does not appear to rely on demographic biases for ADHD prediction, as demonstrated by its poor performance when using only demographic features. This is a desired outcome, as it indicates that additional feature groups provide ADHD-specific information that improves classification.

As was observed, the model’s PR-AUC improves with the addition of CPT features (Demographic + CPT) to the baseline model (Demographic), suggesting that CPT data collected via a smartphone device does provide valuable information for ADHD assessment. However, sensitivity and specificity are lower than studies using machine learning with comparable features on laptop-based CPTs (22, 23, 43). This difference may stem from variations in data collection methods or inherent distinctions in using a smartphone device, such as the holding of the device or interacting through screen taps rather than computer keypresses. The current hypothesis left for future studies to evaluate is if performing a CPT task on a smartphone is harder than in a computerized setting. This way, the separation between the neurotypical and ADHD group could be less distinct (i.e., more commission errors, omission errors, more variation in the reaction time) and the machine learning algorithm has a harder time classifying the cohorts.

The high standard deviation in sensitivity and specificity across runs using the Demographic and CPT feature groups is attributable to the model’s inability to effectively separate ADHD and neurotypical samples. This results in inconsistent threshold-dependent predictions that alternate between favoring the minority or majority class. In contrast, the threshold-insensitive PR-AUC score remains consistent with low variance, as it evaluates performance across all possible thresholds, providing a more reliable metric for models with weak discriminatory power.

Face tracking has previously been shown to be an effective way of using sensor data to extend CPTs with a measure of hyperactivity (11). This is further supported by the significant increase in performance with the addition of the face features (Demographic + CPT + Face).

The motion sensor features are unique to handheld devices and have not been explored previously. The results in this study (Demographic + CPT + Face + Motion) show that data from these sensors can add further information that is useful for ADHD assessments. The addition of the motion feature group led to a significant increase in PR-AUC, and the strong performance of the full feature set (sensitivity: 0.808, specificity: 0.795, PR-AUC: 0.799) highlights the potential of smartphones for ADHD assessment.

Age and sex differences in ADHD are well documented (31, 32, 44), and this study included both adult and child participants as well as males and females (372 adults, 45 children). The model achieved high PR-AUC across age groups, with 0.731 in adults and 0.957 in children, indicating good ability to prioritize true cases despite class imbalance. However, differences were observed in sensitivity and specificity. In adults, performance was balanced (sensitivity 0.752, specificity 0.810), while in children the model showed higher sensitivity (0.987) but lower specificity (0.658). This suggests the model identifies true cases in children effectively but at the cost of more false positives.

These patterns may reflect the small number of children in the test set, which increases variability and can inflate metrics. They may also result from using a single decision threshold across groups, which could be addressed with group-specific thresholds or recalibration. These findings highlight the importance of assessing subgroup performance in imbalanced datasets. While high sensitivity in children reduces the risk of missed cases, it also increases the chance of unnecessary follow-ups. Further research is needed to confirm these results in larger cohorts and to explore age- or sex-specific model adjustments before clinical use.

Future studies aim to validate these results on more cohorts, explore how this approach would work with comorbidities, and if it can be used to measure treatment efficacy. Additionally, integrating various clinical rating scales as features may offer a more comprehensive understanding of patient status, potentially improving model performance in assessing health outcomes.

A key limitation of this study is the potential bias present in the data, which may arise from factors such as sampling methods, or inherent biases in the ADHD diagnosis that the model uses as a ground truth. These biases could affect the model’s ability to generalize to broader populations, and further steps should be taken to mitigate these effects in future analyses.

The relatively small size of the ADHD test set may impact the generalization of the findings. While the results provide valuable insights, a larger test set would allow for more robust validation and increase confidence in the model’s performance across diverse populations. However, the implemented train/test stratification mitigates the potential effect by ensuring both sets contain a similar proportion of classes and sex and age distribution.

This study also did not examine ADHD sub-types, as sub-type labels were not available in the dataset. Further research is needed to evaluate whether smartphone-based assessments perform consistently across ADHD sub-types.

It should be emphasized that this study does not present QbMobile itself, but rather early findings from its development, not a final, validated product. QbMobile is intended as a support tool within the broader, multi-source clinical assessment of ADHD, rather than as a standalone diagnostic test. Accordingly, these findings should be seen as a contribution to the development of complementary assessment tools, not as a replacement for comprehensive clinical evaluation.

## 6 Conclusion

In conclusion, this study is part of the development of a smartphone application (QbMobile) that aims to evaluate the capability of a machine learning algorithm to predict ADHD diagnosis using a smartphone device. We provide a robust machine learning model that is based on a large control group together with an ADHD cohort. The experiments proved that ADHD can be assessed with a high PR-AUC of 0.799, sensitivity of 0.808, and specificity of 0.795 by using a smartphone CPT. The overall strong validation results and the significant performance improvement observed with the addition of smartphone-specific features suggest that smartphone applications have the potential to offer advantages over current computerized ADHD diagnostic tests. These findings highlight the potential of smartphone-based tools to support ADHD assessment as part of a broader diagnostic process.

## Data Availability

The datasets presented in this article are not readily available because they are proprietary to Qbtech AB. Requests to access the datasets should be directed to Simon Larsson, simon.larsson@qbtech.com or Núria Casals, nuria.casals@qbtech.com.
