# Correction: Machine learning on a smartphone-based CPT for ADHD prediction

**DOI:** 10.3389/fpsyt.2025.1771553

**Published:** 2026-01-06

**Authors:** Núria Casals, Simon Larsson, Mikkel Hansen

**Affiliations:** Medical Department, Qbtech AB, Stockholm, Sweden

**Keywords:** ADHD, machine learning, CPT, smartphone, mobile, motion sensor, face tracking, AI

In the **Abstract**, paragraph 3, a specificity value was missing due to a typographical error. This paragraph read as, “A total of 952 neurotypical individuals and 292 unmedicated ADHD patients were part of the study. The best performing model combines all feature groups by a sensitivity of 0.808, specificity of blue and area under the precision-recall curve (PR-AUC) of 0.799, with a considerable performance increase due to the phone sensor features addition. Results did not differ significantly by age group (6–11 and 12–60 years old) and sex.” The corrected paragraph should read as, “A total of 952 neurotypical individuals and 292 unmedicated ADHD patients were part of the study. The best performing model combines all feature groups by a sensitivity of 0.808, specificity of 0.795 and area under the precision-recall curve (PR-AUC) of 0.799, with a considerable performance increase due to the phone sensor features addition. Results did not differ significantly by age group (6–11 and 12–60 years old) and sex.”

The original version of this article has been updated.

