# Supplementary material for: Machine learning on a smartphone-based CPT for ADHD prediction
Source: Front Psychiatry. 2025 Nov 7;16:1564351. doi: 10.3389/fpsyt.2025.1564351 (PMC12634579; doi:10.3389/fpsyt.2025.1564351)
Supplement: Supplementary file 1 [file DataSheet1.pdf]

Supplementary Material

1 ERROR ANALYSIS OF CPT ERROR PARAMETERS

The addition of Face and Motion features improved the performance of the model, enabling it to correctly classify cases that would have been misclassified when relying solely on CPT features. To illustrate this, a single model run was taken to perform an error analysis, focusing on the classification power of CPT error parameters.

Table S1 shows the differences in the model parameters between the Demographic + CPT model and the Demographic + CPT + Face + Motion model. Specifically, it compares individuals who were correctly classified by the Demographic + CPT model and those who were misclassified by that model but correctly classified when Face and Motion features were added. It includes key CPT parameters such as commission errors, omission errors, and overall error rate, reported as average percentages across individuals within each group.

Demographic+CPT	Demographic+CPT+Face+Motion	N	Commission	Omission	Error rate
TP	-	47	8.03	16.13	8.88
TN	-	106	1.45	5.27	2.60
FP	FP $\rightarrow$ TN	52	5.51	6.91	5.33
FN	FN $\rightarrow$ TP	7	2.22	5.57	2.81

Table S1. CPT error metrics (commission error, omission error, and error rate) reported as averages for predictions originally made by a model trained on Demographic + CPT, including only false positives and false negatives that were corrected by the Demographic + CPT + Face + Motion model, with true positives and true negatives included as reference.

It was found that some cases of ADHD with good CPT performance were misclassified when using only CPT features, but were correctly identified when Face and Motion features were included. Similarly, some neurotypical cases with poor CPT performance were incorrectly labeled based only on CPT data, but were correctly classified when incorporating all feature groups. The average commission error among True Positives correctly identified by the Demographic + CPT model is 8.03. In contrast, the complete model (Demographic + CPT + Face + Motion) correctly identifies 7 additional True Positives, with a lower average commission error of 2.22. Similarly, the average commission error among True Negatives correctly classified by the Demographic + CPT model is 1.45, while the complete model identifies 52 additional True Negatives, with a higher average commission error of 5.51.

2 ERROR ACROSS FEATURE GROUPS

We evaluated the impact of sequentially adding different groups of features on the performance of our binary classification model. The four groups of features (Demographics, CPT, Face and Motion) were introduced step by step, allowing us to track how each group contributed to improving the model's ability to correctly predict the target labels.

Figure S1 is a Sankey diagram that illustrates the flow of predictions as more feature groups were added. The diagram shows that with each additional feature group, the number of correctly classified instances consistently increased. This highlights how each group contributed additional predictive power, enabling the model to make more accurate decisions.

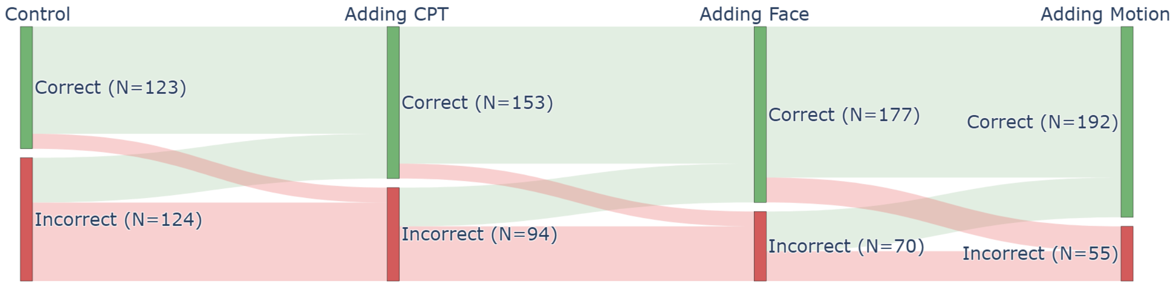

Figure S1. Correct vs. incorrect predictions across feature group additions, visualized using a Sankey diagram.
